# The status of drowning prevention and control in the region of the Americas

**DOI:** 10.1186/s40621-025-00601-0

**Published:** 2025-08-25

**Authors:** Alessandra F. Senisse-Pajares, Caroline Lukaszyk, Ricardo Pérez-Núñez

**Affiliations:** 1 PAHO – Pan-American Health Organization, 525 23rd Street NW, Washington, DC USA; 2 WHO – World Health Organization, Geneva, Switzerland

**Keywords:** Drowning prevention, Injury, Risk, Water safety, Americas, Policy

## Abstract

Drowning is a major, yet preventable, public health issue causing an estimated 300,000 deaths globally in 2021. This study assessed the current state of drowning prevention efforts in the Americas, examining the presence of governance structures, policies, legislation and the implementation of key interventions to reduce drowning risks. Data were collected from 26 countries in the region through a World Health Organization-led initiative using a structured questionnaire and national consultations. Of the 26 countries studied, only two (8%) reported having a government-led national drowning prevention strategy, and 65% of countries collected information on drowning mortality data through civil registration systems. Despite a wider presence of disaster risk management programs and public awareness campaigns which address drowning risks, substantial gaps remain in interventions aimed at preventing child drowning, including swim skills and water safety training. These findings underscore the urgent need for standardized strategies, improved data systems, and stronger cross-sectoral collaboration to reduce drowning deaths in the Americas.eat

## Introduction

Drowning is a major yet preventable global health challenge, responsible for an estimated 300,000 deaths during 2021. It ranks as the fourth leading cause of mortality among children aged 5–14 years globally, and is the seventh leading cause of death for the same age group in the Americas [1]. While the global drowning mortality rate is 3.8 per 100,000 population, the Region of the Americas reports a lower rate of 1.6 per 100,000, representing an estimated 17,000 lives lost during 2021 and an estimated 950 thousand healthy life years lost (DALY) [1]. In the Americas, two vulnerable age groups are most affected: children aged under five years and adults aged 70 years and above (with drowning mortality rates of 2.2 and 1.9 per 100,000 population, respectively). Significant disparities in drowning mortality rates exist among countries in the Americas, with unintentional drowning having the highest associated index of disparity among all external causes of injuries [2]. 

Despite decades of work by advocates and organizations, drowning prevention has remained a low priority on the global policy agenda until recent years. In 2021, this changed with the adoption of the first-ever United Nations General Assembly (UNGA) Resolution on Drowning Prevention (A/RES/75/273), endorsed by all 193 UN Member States. The resolution recognized drowning as a preventable public health threat, emphasizing the availability of scalable and low-cost interventions, and called for urgent, coordinated action from governments, organizations, and communities worldwide. It also designated the World Health Organization (WHO) as the lead agency for global efforts in drowning prevention [3]. 

Building on this momentum, the World Health Assembly (WHA) adopted Resolution WHA76.18 in 2023 to accelerate global action on drowning prevention. This resolution reinforces and expands previous commitments, urging Member States to assess their national drowning burden, develop comprehensive and multisectoral prevention strategies, and integrate these strategies into broader health, safety, and development policies. Recognizing the importance of accurate data to inform effective action, the resolution mandated WHO to conduct a global situational assessment on drowning to inform the development of evidence-based policies [4]. This assessment culminated in the publication of the first Global status report on drowning prevention in December 2024.

While WHO had previously issued technical guidance and recommendations to support drowning prevention efforts [5–7], these resolutions marked a turning point in the global recognition of drowning as a critical and preventable public health challenge. They laid the foundation for coordinated action at global, regional, and national levels. Central to this process was the need to document the status of drowning prevention efforts worldwide, to share this information thorough establishing a global database, and to provide a baseline for monitoring progress over time.

The objective of this paper is to analyze the data collected for the Region of the Americas as part of the Global status report on drowning prevention 2024 [1], to better understand the regional burden of drowning, assess the presence of governance structures, policies, legislation and key interventions that reduce drowning risks, and to identify priority actions to advance drowning prevention efforts in the region.

## Methodology

### Design and study population

This article draws on data collected between July 2023 and October 2024 which contributed to development of the Global status report on drowning prevention 2024 [1]. All countries were invited to contribute, with 26 countries in the Americas voluntarily participating in this global effort. These included 25 of the 35 Member States, and one territory of the Pan American Health Organization (PAHO). Antigua and Barbuda, Barbados, Grenada, Haiti, Nicaragua, Saint Kitts and Nevis, Saint Lucia, Saint Vincent and the Grenadines, Trinidad and Tobago, and Venezuela chose not to participate in this global effort. The participating countries represented 71.4% of the Member States in the region and account for 95% of the population of the Americas.

### Data collection

To support evidence-based decision-making, WHO, in collaboration with approximately 20 global experts, developed a comprehensive questionnaire to be completed through a national consultation process. The questionnaire was designed to collect important information, along with supporting documentation, on three key areas relevant to drowning prevention: (1) management and coordination; (2) policies and legislation; and (3) key community interventions. Within these three domains, the questionnaire covered a broad range of topic areas, including:

- Existence of a designated government focal point for drowning prevention.
- Existence of national or subnational drowning prevention strategies or plans.
- Multisectoral involvement and mechanisms for stakeholder coordination.
- Data collection practices and sources (e.g., CRVS, health, police, maritime).
- Availability of public awareness campaigns (e.g., communicating risks associated with alcohol use and drowning, promoting child supervision).
- Laws and policies on swimming pool fencing, water transport safety, lifejacket use, and alcohol consumption regulations.
- Implementation of community-based interventions, such as swimming and water safety lessons through schools, supervised childcare programs, installation of barriers near water, disaster warning systems, flood risk management programs, boating safety, and search and rescue services.

This simplified list of covered topics is intended to help readers navigate the scope of the results presented in this report.

The questionnaire included a combination of open-ended questions aimed at capturing national narratives and contextual insights, as well as structured multiple-choice and dichotomous (yes/no) fields to assess the presence of specific strategies, actors, legal frameworks and programs. Matrix-style tables were used to document the presence, reach, and implementation status of interventions, while checkbox formats allowed respondents to indicate relevant sectors and organizations involved in drowning prevention. The questionnaire requested multisectoral inputs and submission of supporting documentation such as national plans, legislative texts, and mortality data tables to validate responses and strengthen data completeness.

Each participating country designated a National Data Focal Person (NDFP) responsible for coordinating the completion of the questionnaire. Prior to data collection, all NDFPs received training and technical guidance from their assigned Regional Data Focal Point (RDFP), aimed at ensuring the standardized application and interpretation of the questionnaire across countries. The NDFPs led a national consultation process, engaging six to ten respondents from relevant government sectors and non-government organizations to contribute to the data collection. Respondents were identified based on their institutional responsibilities related to drowning prevention and typically included health, tourism, disaster response, police, lifeguards, coast guard, non-governmental organizations (NGOs) such as the Red Cross, lifesaving organizations and swimming associations. Stakeholder engagement in drowning prevention was demonstrated both through participation in the consultation process and the documented roles of stakeholders in national prevention activities.

Each respondent independently completed the questionnaire and subsequently participated in a national consensus meeting. During this meeting, respondents reviewed and harmonized their questionnaire inputs to generate one final submission for their country, which the NDFP shared with the RDFP at PAHO. The RDFP reviewed the submitted responses to identify and address any inconsistencies before entering the data into an online global data platform. The information was then further validated by the global coordinator at WHO Headquarters.

In addition to questionnaire responses, participating countries were asked to submit specific national policies, strategies, and legislation relevant to drowning prevention. A multilingual lawyer proficient in Spanish, English, and Portuguese conducted a detailed review and analysis of documents provided by countries to ensure accuracy and contextual understanding (see Sect. 2 on policies and legislations). Country information within the database was shared with each respective NDFP for review, approval, or to request additional supporting documentation as necessary.

This comprehensive approach not only provided a detailed assessment of the current state of drowning prevention in each country but also provided an opportunity to share WHO resources on best practice approaches for reducing drowning risks. Through seeking multi-sector collaboration towards one final country questionnaire submission, a diverse group of stakeholders had the opportunity to convene specifically on the topic of drowning prevention.

### Analysis

This analysis is based on the final database approved by participating governments from the Region. A descriptive analysis (frequencies) of relevant variables and indicators was performed at the regional and subregional level. To provide a better overview of the Region, participant countries were categorized into four broad groups:

- North America (2): United States of America and Canada.
- Mesoamerica (7): Belize, Costa Rica, El Salvador, Guatemala, Honduras, Mexico, and Panama.
- Caribbean (6): Bahamas, Bermuda, Cuba, Dominica, Dominican Republic, and Jamaica.
- South America (11): Argentina, Bolivia, Brazil, Chile, Colombia, Ecuador, Guyana, Paraguay, Peru, Surinam, and Uruguay.

To complement the quantitative analysis, a content review of national and subnational legislation related to drowning prevention was conducted using the legal documents submitted by countries. This review was carried out by a WHO appointed legal expert and was guided by the evidence-based policy recommendations outlined in WHO’s Preventing drowning: an implementation guide. The expert systematically examined the submitted laws and regulations to assess the existence, scope, and specificity of legal frameworks in key drowning prevention domains, including swimming pool fencing, disaster risk reduction, and regulations concerning the seaworthiness and operation of domestic passenger vessels. Each document was analyzed to determine whether legislation was in place, at what level (national or subnational), and whether core attributes were included—such as enforcement mechanisms, technical specifications, and applicability to specific settings or populations. A combination of binary coding (e.g., presence or absence of legislation) and qualitative notes was used to enable comparative analysis across countries. This approach allowed for a comprehensive understanding of both policy frameworks and practical interventions, contributing to a robust evaluation of regional progress and challenges in drowning prevention.

## Results

Table 1 presents a list of the key indicators for drowning prevention documented at regional and subregional levels.

**Table 1:** Percentage of countries meeting key indicators on drowning prevention in the americas, by subregion

Area	Indicator	% North America (*n* = 2)	% Mesoamerica (*n* = 7)	% Caribbean (*n* = 6)	% South America (*n* = 11)	% All Region (*n* = 26)
Management and coordination	Countries with a government nominated national focal person or sector/department for drowning prevention	0% (0)	57% (4)	50% (3)	36% (4)	42% (11)
	Countries with a national coordination mechanism for drowning prevention including the government	100% (2)	29% (2)	0% (0)	27% (3)	27% (7)
	Countries who have a national drowning prevention strategy	100% (2)	29% (2)	17% (1)	9% (1)	23% (6)
	Countries who have a national drowning prevention strategy with specific and measurable mortality reduction targets and timeframes	50% (1)	14% (1)	0% (0)	0% (0)	8% (2)
	Countries that capture drowning deaths through their civil registration and vital statistics registers	100% (2)	57% (4)	67% (4)	64% (7)	65% (17)
	Countries that routinely collect at least one source of sufficiently detailed drowning data (with level of detailed recorded including age, sex, activity at time of drowning and type of water body)	100% (2)	86% (6)	100% (6)	55% (6)	77% (20)
	Countries with research/academic institutions actively contributing to reducing the likelihood of drowning	100% (2)	0% (0)	0% (0)	55% (6)	31% (8)
	Countries running national mass media communication campaigns with a specific focus to prevent drowning	100% (2)	57% (4)	33% (2)	18% (2)	38% (10)
Policies and legislations	Countries that have a dedicated national strategy, policy or plan for disaster risk management that prioritizes drowning risk reduction	50% (1)	29% (2)	17% (1)	27% (3)	27% (7)
	Countries with national laws that set out minimum safety requirements for the seaworthiness and operation (e.g. operator certification, passenger capacity etc.) of domestic passenger vessels	100% (2)	86% (6)	100% (6)	91% (10)	92% (24)
	Countries with national regulations which make lifejacket use compulsory during recreational boating and/or on passenger transport vessels.	0% (0)	71% (5)	50% (3)	82% (9)	65% (17)
	Countries with national laws that require the use of fencing to exclude unsupervised child access to public and or private swimming pools	0% (0)	14% (1)	33% (2)	18% (2)	19% (5)
	Countries that regulate alcohol consumption and/or sales from vendors operating in close proximity to public waterbodies such as beaches and public swimming pools at the national level	0% (0)	29% (2)	17% (1)	27% (3)	23% (6)
Community interventions	Countries who implement interventions promoting installation of physical barriers to control the access of children to water at the national level	0% (0)	14% (1)	33% (2)	27% (3)	23% (6)
	Countries teaching swimming and water safety to children through schools as part of routine national curriculum	0% (0)	0% (0)	17% (1)	9% (1)	8% (2)
	Countries who provide safe places for pre-school children with structured arrangements for childcare provided by trained adults at the national level	0% (0)	0% (0)	33% (2)	18% (2)	15% (4)
	Countries with a nationally operating, dedicated search and rescue service, which is fully operational	100% (2)	86% (6)	67% (4)	64% (7)	73% (19)
	Countries that provide lifeguard services at all designated public swimming venues at the national level	0% (0)	57% (4)	50% (3)	36% (4)	42% (11)
	Countries that offer regular and financially accessible programmes with accredited trainers to train bystanders in safe rescue and resuscitation with national coverage	50% (1)	29% (2)	17% (1)	27% (3)	27% (7)
	Countries with advanced national cyclone/flood/tsunami warning systems in place and are operational	50% (1)	86% (6)	100% (6)	64% (7)	77% (20)
	Countries who provide freely available weather alerts with information for safety on or near waterbodies, or in direct relation to flooding risks at the national level	50% (1)	86% (6)	100% (6)	91% (10)	88% (23)
	Countries with national community resilience programmes that include drowning prevention measures for example basic rescue, first aid, swim skills and other drowning prevention awareness	0% (0)	57% (4)	33% (2)	9% (1)	27% (7)
	Countries who undertake national efforts to build community resilience to disasters and to manage flood risks	50% (1)	86% (6)	67% (4)	82% (9)	77% (20)

### Management and coordination

The questionnaire collected information on national leadership and coordination for drowning prevention, assessing the existence of designated government focal points, presence of strategies or plans, the breadth of sectoral involvement, and existing mechanisms for intersectoral coordination. The results below present a regional and subregional overview of these components.

#### Leadership

In the Americas region, 42% of participating countries reported having a government-designated national focal point for drowning prevention. Among these, only two countries, Mexico and Jamaica, had a focal point within the health sector (Table 2). Just over half of all participating countries (54%) reported not having a designated national focal point, while only country, Belize, indicated that this information was unknown.

**Table 2:** Government-designated focal points in the Americas

Country	Drowning prevention focal point	Sector
Brazil	Civil Defense and Firefighters	Disaster risk reduction
Chile	Chilean Navy	Military/Coastguard
Colombia	National Disaster Risk Management Unit and the Colombian Navy	Military/Coastguard
Costa Rica	National Commission for the Prevention and Attention of Drownings, which operates under the National Emergency Commission (CNE)	Disaster risk reduction
Cuba	The General Aeronautical and Maritime Coordination Center for Search and Rescue as well as the Aeronautical Coordination Center and the Maritime Coordination Center, of the Ministry of Transportation	Transportation
El Salvador	Emergency Operations Center (COE) part of the Ministry of Governance and Territorial Development	Disaster risk reduction
Honduras	Permanent Commission of Contingencies (COPECO) that leads the National Committee for the Prevention of Mass Mobilizations (CONAPREM).	Disaster risk reduction
Jamaica	Emergency Medical Services (EMS) are part of the Emergency, Disaster Management & Special Services Branch of the Ministry of Health and Wellness (MOHW)	Health
Mexico	Technical Secretariat of the National Council for Accident Prevention (STCONAPRA) of the Ministry of Health	Health
Uruguay	Interagency Water Safety Committee under the leadership of the National Emergency System (SINAE)	Disaster risk reduction
Bermuda	Water Safety Council of the Ministry of Tourism Development and Transport	Tourism

#### National drowning prevention strategies

During the consultation process, countries were asked whether they had a national or subnational strategy, policy, or plan specifically addressing drowning prevention. Analysis revealed that 23% of participating countries reported to have a national drowning prevention strategy or plan. However, only strategies developed by Canada and Mexico set specific, measurable targets for reducing drowning mortality with defined timeframes, and only strategies developed by Brazil and Panama had been evaluated to assess their effectiveness.

Drowning prevention strategy development and implementation was led by government agencies in Mexico and Panama, and non-governmental entities in Brazil, Canada, and the United States. Notably, Cuba reported a whole-of-society approach for strategy development and implementation, which involved both government and non-governmental organizations.

Additionally, Uruguay and Chile indicated that national strategies or plans were under development and/or pending formal government endorsement.

#### Sectors involved and coordination

**Fig. 1:**
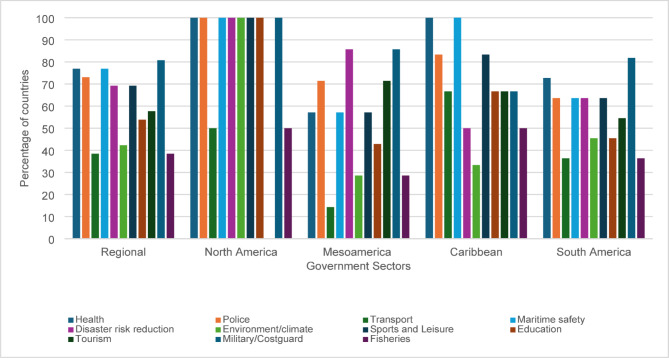
Government sectors involved in drowning prevention in the Americas

Analysis revealed substantial multisectoral contributions towards drowning prevention efforts across the Region, particularly including sectors such as health, police, maritime safety, and disaster risk reduction (Fig. 1).

The highest levels of engagement were reported from the military/coastguard (81% of countries), followed by maritime safety (78% of countries), health (78% of countries) and police (73% of countries). Disaster risk reduction and sports and leisure sectors were also notably active, contributing to drowning prevention efforts in 69% of countries.

However, subregional differences were observed. North America reported the highest level of intersectoral engagement in national drowning prevention efforts, while Mesoamerica and South America reported lower overall intersectoral engagement. In Mesoamerica, sectors responsible for transport, fisheries and environment/climate were reported to have the lowest levels of engagement in drowning prevention. A similar trend was observed in South America. The Caribbean reported strong involvement in drowning prevention from maritime safety and health, but lower participation from sectors responsible for environment/climate and disaster risk reduction. These findings highlight notable disparities in sectoral engagement on drowning prevention across subregions.

**Fig. 2:**
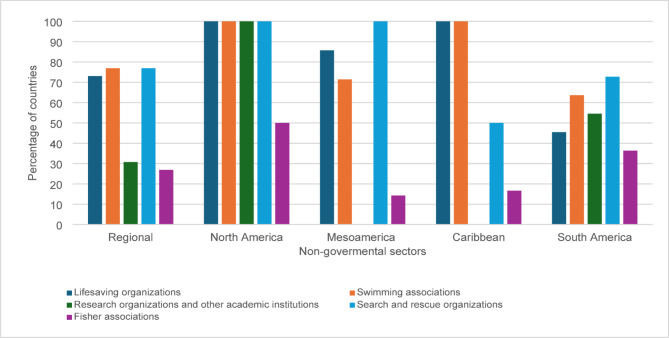
Non-governmental organizations involved in drowning prevention in the Americas

As shown in Fig. 2, key non-governmental stakeholders involved in drowning prevention include swimming associations and search and rescue organizations, both reported actively working towards water safety in 77% of countries. Lifesaving associations were reported to contribute to national drowning prevention efforts in 73% of countries, while research organizations and other academic institutions were reported to make contributions in 31% countries.

Despite the involvement of diverse stakeholders in national drowning prevention efforts, only 27% of countries reported to have a formal coordination mechanism for drowning prevention involving both governmental and non-governmental actors. One country (Cuba) reported coordination efforts exclusively among government stakeholders.

#### Data and research

To assess national data collection efforts, countries were asked whether they capture drowning deaths through their Civil Registration and Vital Statistics (CRVS) systems, whether the data collection is ongoing, compiled at a national level, and the level of detail captured.

Results show that 65% (17 countries) collect drowning mortality data through CRVS systems on a permanent, ongoing basis. All countries collecting drowning data through CRVS reported capturing the age and sex of drowning victims. However, among these countries, only 71% recorded the type of waterbody in which the drowning event occurred and just 48% documented the activity at the time of drowning (Table 3). Additionally, only 54% of countries reported to compile drowning data at the national level. Across the Region, five countries reported high standards for drowning death data collection practices, with ongoing, national-level data systems capturing key variables such as victim age, sex, activity at the time of drowning, and waterbody type.

**Table 3:** Number of countries with drowning death data through civil registration and vital statistic in the Americas

Drowning data are collected through civil registration and vital statistics registers (CRVS)	17 (65%)
Data are collected in an ongoing permanent manner	17 (65%)
Data are compiled and reported at the national level	14 (54%)
**Of the 17 countries which collect drowning death data through CRVS:**	
Age is recorded	17 (100%)
Sex is recorded	17 (100%)
Activity at time of drowning is recorded	8 (48%)
Type of water body is recorded	12 (71%)

Countries were asked whether drowning data are collected by various sectors, including health, police, maritime safety, disaster risk management, occupational safety, sport and leisure, transport, and tourism. A majority of countries reported collecting data from at least three or more sectors, showcasing potential for cross-sectoral data integration. However, the frequency of data collection and level of detail captured varied significantly across sectors.

**Fig. 3:**
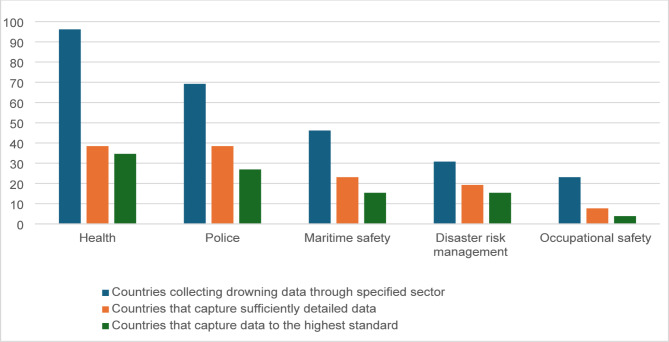
Percentage of countries that collect drowning death data through different sectors in the Americas. Note: “Sufficiently detailed” refers to sources that capture four specific indicators for drowning deaths: age, sex, activity at time of drowning and type of water body. “Highest standard” denotes sources (1) collect drowning death data in an ongoing and permanent manner, (2) compile and report data at the national level, and (3) capture sufficient detail for each drowning death, including the age, sex, activity at time of drowning and type of water body

Health and police sectors were reported to be most active in collecting drowning data in the region. This was followed by maritime safety (46%) and disaster risk management (31%) (Fig. 3). Notably, 77% of countries indicated collecting sufficiently detailed data through health and police sectors, including variables such as age, sex, activity at the time of drowning, and type of waterbody.

Despite these efforts, only 35% of health and 27% of police sectors met the criteria for high-standard data collection, defined as ongoing, national-level systems capturing detailed drowning related variables. Other sectors, such as tourism, sport and leisure, and transport, reported limitations in data collection. Additionally, only 19% of countries reported CRVS systems that met the criteria for high-standard data collection.

#### Raising awareness

Countries were asked about three topics of public awareness campaigns aimed at reducing drowning: (1) national mass media communication campaigns focused on drowning prevention, (2) messaging on the dangers of alcohol consumption around water, and (3) campaigns emphasizing the importance of child supervision around water.

**Fig. 4:**
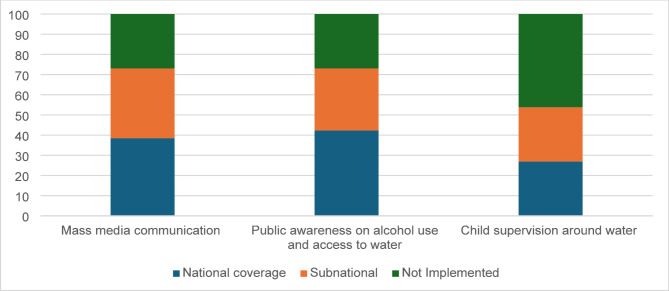
Percentage of countries with public awareness interventions for drowning prevention in the Americas

Approximately 38% of countries reported implementing national mass media campaigns addressing drowning prevention, while 26% indicated they had not conducted such campaigns. To raise awareness on the dangers of alcohol use around water, 42% of countries reported implementing national public awareness campaigns, with an additional 31% conducting campaigns at the subnational level. To promote the importance of effective child supervision around water, 27% of countries reported implementing national campaigns, while 46% indicated they either had no initiatives or were unsure of their existence (Fig. 4).

### Drowning prevention National policies and legislations

#### Disaster risk management policy

In the Americas, 27% of participating countries reported dedicated disaster risk management policies that specifically address drowning risks. Brazil reported the existence of such policies at subnational levels.

#### Legislation for safety of passenger water transport vessels

**Fig. 5:**
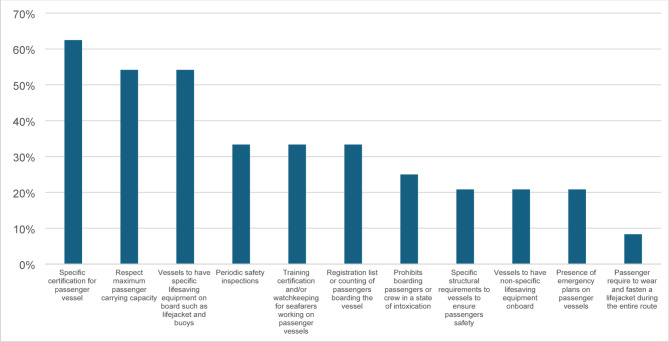
Percentage of countries with key safety attributes in their national regulations for water-transport vessels in the Region of the Americas. Note: Denominator to calculate percentages is 24 as legislations from Guyana and Guatemala were not included here

In the Americas, 92% of participating countries reported having national regulations in place to ensure water transport vessel safety. This corresponded to 24 pieces of legislation. Content analysis of submitted legislation revealed significant variation in both content and comprehensiveness of existing laws. Eleven key safety attributes were identified, with varying levels of inclusion across legislation from different countries (Fig. 5). Specific certification requirements for passenger vessels were present in 63% of national laws. Regulations on maximum passenger capacity as well as the presence of essential lifesaving equipment such as life jackets and buoys, were each included in 54% of the laws reviewed. Provisions for periodic safety inspections, training and certification of seafarers, and mandatory passenger registration lists were found in 33% of national laws. Notably, requirements for passengers to wear lifejackets were frequently excluded, reported in fewer than 10% of national laws, despite this being a critical drowning prevention measure.

#### Lifejacket use

In contrast to the legislative review findings, 65% of countries reported having national legislation mandating lifejacket use. The questionnaire did not disaggregate responses by type of vessel, user group, or enforcement mechanisms, which likely leads to the observed discrepancies between legislation mandating lifejacket use in all contexts versus a more specific mandate for lifejacket use while travelling on water transport vessels.

#### Swimming pool fencing

Countries were asked about the presence of national or subnational laws mandating fencing to prevent unsupervised child access to public and/or private swimming pools. The presence of such legislation remains limited, with only nine countries (35%) in the Americas reporting any laws related to swimming pool fencing (Table 4). Among these, four countries reported legislation at the subnational level. Only two countries reported laws requiring fencing around both public and private pools, while the remaining seven had legislation specific to public pools only.

**Table 4:** Countries with swimming pool fencing legislation

Country	Level of law	Law covers private pool fencing	Law covers public pool fencing	Fencing height required	4-sided fencing required	self-closing/ self-latching gate required
Argentina	Sub-national	No	Yes	Yes	Yes	Yes
Brazil	Sub-national	No	Yes	No	No	No
Chile	National	No	Yes	No	No	No
Colombia	National	No	Yes	No	No	No
El Salvador	National	No	Yes	Yes	No	No
Jamaica	National	No	Yes	No	No	No
Mexico	Sub-national	No	Yes	No	No	No
United States of America	Sub-national	Yes	Yes	Yes	Yes	Yes
Bermuda	National	Yes	Yes	Yes	Yes	Yes

Content analysis of submitted laws revealed varying degrees of content and comprehensiveness. Among the countries with pool fencing legislation, four specify minimum fence height requirements, three mandate four-sided fencing, and three require self-closing or self-latching gates. Only two countries reported legislation that includes all key attributes for effective pool fencing, and just one country reported such legislation at the national level.

#### Alcohol regulations

Regulations restricting alcohol consumption near public waterbodies remain limited in the Region. Only 23% of countries reported having national legislation regulating alcohol consumption in close proximity to water, while 54% indicated the absence of any legislation of this nature.

### Community interventions for drowning prevention

#### Child drowning prevention interventions

##### Barriers near water

**Fig. 6:**
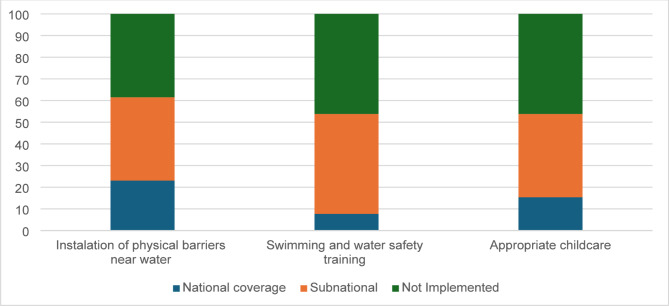
Percentage of countries implementing children’s drowning prevention interventions in the Americas

In the Region of the Americas, 23% of countries reported having national programs that promote the installation of physical barriers to prevent unsupervised children from accessing waterbodies. At the subnational level, 38% reported similar programs, although these initiatives are often limited in reach (Fig. 6).

##### Swimming and water safety skills training

Only 8% of countries reported having national programs to integrate swimming and water safety training into school curricula. However, 46% of countries indicated such programs being delivered at the subnational level (Fig. 6).

##### Childcare

Only 15% of countries reported having national programs that provide structured, supervised childcare, offering safe spaces for preschool-aged children away from water hazards (Fig. 6). In contrast, 46% of countries reported having no such initiatives or were unaware of their existence.

#### Interventions specific to disaster risk reduction

##### Disaster warning systems

**Fig. 7:**
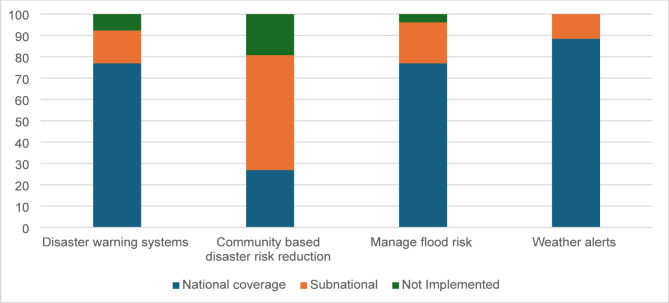
Percentage of countries with disaster risk reduction interventions in the Americas

Countries were asked whether disaster warning systems had been implemented to provide timely alerts, particularly in areas at high risk of water-related disasters. A total of 77% of countries reported having disaster warning systems with national coverage, while 15% reported systems implemented at the subnational level (Fig. 7).

##### Freely available weather alerts 

Weather alert systems were among the most widely implemented measure that supports drowning prevention in the Region, with 88% of countries reporting operating weather alert systems at the national level (Fig. 7).

##### Flood risk management and community disaster resilience/risk reduction (first responder training)

Flood risk management efforts appear strong across the Region, with 77% of countries reporting national coverage. However, community-based disaster risk reduction programs, including first responder training, show greater variability. While 27% of countries reported having national programs, 54% reported relying primarily on subnational initiatives.

#### Interventions specific to boating and public safety 

##### Boating safety

National coverage of boating safety regulations was reported by 73% of participating countries. However, this percentage was notably lower in Caribbean countries (33%) compared to other subregions (see Table 1).

##### Public safety interventions

**Fig. 8:**
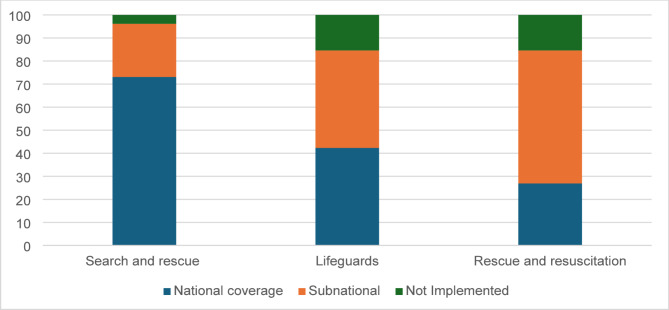
Percentage of countries implementing public safety interventions for drowning prevention in the Americas

In the Americas, 73% of countries report having fully operational national search and rescue services, while an additional 23% reported to provide these services at the subnational level, prioritizing coverage in high-risk areas (Fig. 8). Encouragingly, only 4% of countries reported having no search and rescue services in place.

Lifeguard services at designated public swimming areas are available at the national level in 42% of countries. However, 15% of countries reported having no lifeguard services (Fig. 8).

Rescue and resuscitation training programs were reported to be implemented at the national level in 27% of countries, with 58% of countries relying on subnational initiatives. Despite their critical role in improving survival rates following a drowning event, 15% of countries reported having no such programs (Fig. 8).

## Discussion

Drowning remains a significant cause of death in the Americas, particularly among children under five years of age, despite being largely preventable. This first regional assessment provides an opportunity to identify key gaps in the implementation of best practice for drowning prevention, thereby supporting context-specific and needs-based planning. In addition, the assessment establishes a foundation for ongoing monitoring and evaluation of national and regional progress in implementing relevant policies, strategies and specific interventions. Sustained progress will require maintaining existing momentum and fostering collaboration across government sectors, UN Agencies, civil society, and donors to strengthen and support national efforts [8]. 

The Americas currently faces critical gaps in strong governance and leadership for drowning prevention. Only 11 countries (42%) reported to have a government-appointed national focal point, while just 8% of countries have government-led drowning prevention plans in place. National focal points are essential in providing leadership, ensuring accountability, and facilitating coordinated action. Encouragingly, multiple sectors are engaged in drowning prevention across the Region, reflecting a intersectoral approach to the issue and emphasizes the importance of collaborative efforts to enhance safety measures and awareness. The significant involvement of non-governmental stakeholders further demonstrates broad engagement across sectors. Each government and non-governmental entity fulfil a unique and critical role in promoting water safety and reducing drowning incidents. However, only 27% of countries reported having formal coordination mechanism that involves both governmental and non-governmental actors, potentially limiting effective collaboration and coordination. Strong intersectoral coordination, coupled with active involvement of civil society, is crucial for strengthening community-based strategies and ensuring the sustainability and scalability of prevention efforts [9]. 

Data collection remains a major challenge towards fully understand the burden and context of drowning in the Americas. Only 65% of countries reported collecting drowning death data through civil registration and vital statistics (CRVS) systems. Incomplete or inconsistent data challenges the ability to identify at-risk populations and specific circumstances leading to increased drowning risk, thereby limiting the development of targeted, evidence-based interventions. For example, an observational study of fatal and non-fatal drownings in Mexico revealed that drowning deaths peak during specific months, days, and times, with children under five years old particularly affected [10]. These findings highlight opportunities to direct resources for maximum impact. Replicating similar studies across the Region could significantly improve understanding of drowning risks patterns and support the design of more effective, evidence-based strategies for the most affected populations.

Additionally, another observational study analyzed home-based risks for unintentional injuries in low-income Mexican households with children under five years of age. The study found that one in four homes where children aged 1–4 years lived used unsafe water storage practices, which is a well-established and persistent risk factor for unintentional drowning in this age group [11–13]. Strengthening prevention strategies—such as safe household water storage practices—and expanding public education efforts are critical to reducing drowning risk among vulnerable populations. These findings reinforce the importance of improved data collection and the implementation of evidence-based interventions to reduce drowning across the Americas.

WHO estimates that increased investment in drowning prevention could save the lives of up to 774,000 children, prevent nearly one million non-fatal drownings, and avert over US$400 billion in economic losses due to premature deaths and morbidity by 2050 [14]. These significant estimates are based on the large-scale implementation of two evidence-based drowning prevention interventions: providing daycare for pre-school children and teaching basic swimming skills to school-age children. With a return of US$9 for every dollar invested, the economic and societal benefits of prioritizing drowning prevention are substantial [14]. This underscores the urgent need for governments and organizations to implement comprehensive, multisectoral strategies, allocate dedicated resources, and establish national leadership structures such as designated focal points to ensure sustained and effective drowning prevention efforts.

The Region also lags in enacting legislation and policies that reduce drowning risk. Only nine countries (35%) reported legislation mandating swimming pool fencing, and just 8% reported to integrate swimming lessons into national school curricula. Given that children aged under five years bear the greatest burden of drowning in the Americas, this policy gap highlights the urgent need for a multifaceted approach to drowning prevention focused on child safety. This approach should include equitable access to formal swimming and water safety instructions in schools; legislation requiring physical barriers to restrict access to waterbodies such as swimming pools; and strengthened supervision for children under five years of age. Combined with community education, these interventions form the foundation of an effective framework to address childhood drowning across the Region [15]. 

## Conclusion

While significant challenges persist, this assessment highlights considerable opportunities for coordinated action to reduce drowning deaths in the Americas. Multisectoral collaboration, community-based initiatives, and evidence-based policies are critical to advancing national and regional efforts. The results provide a baseline for monitoring progress and emphasize the need for countries to implement comprehensive national drowning prevention plans and strengthen data systems. These efforts must be supported by increased public awareness, investment in proven interventions, and targeted to the most vulnerable populations. By addressing these priorities and fostering collaboration across sectors and organizations, the Region of the Americas can make meaningful progress in preventing drowning and sustaining long term impact [8]. 

### Limitations

The findings presented are subject to several methodological limitations. Much of the data was obtained through country self-report, which may introduce inconsistencies in interpretation, completeness, and the scope of internal consultation processes. Although national focal points received training and technical guidance to promote standardized application of the questionnaire, the extent and quality of multisectoral engagement likely varied across countries, potentially affecting the reliability and comprehensiveness of responses. In some instances, relevant programs or subnational initiatives may not have been captured if they were not well known to national respondents or formally documented.

The analysis of legislation was limited to the documents submitted by countries, which varied in level of detail, legal scope, and specificity. Documents were reviewed in their original languages, but differences in formatting, legal terminology, and document structure sometimes constrained cross-country comparisons. In some cases, relevant legislative measures may exist but were not submitted or were embedded in broader legal frameworks that were not identified during the consultation process. Additionally, the questionnaire did not systematically capture information on enforcement mechanisms, the stage of implementation, or whether legislation applied to specific contexts (e.g., public vs. private settings or recreational vs. commercial activities). These factors should be considered when interpreting the results and comparing national efforts across the Region.

## Data Availability

Most of the data is available at https://www.who.int/teams/social-determinants-of-health/safety-and-mobility/global-report-on-drowning-prevention (and more specifically in the WHO Drowning Prevention Data app); other information might be shared upon request to corresponding author.

## Declarations

### Data sharing statement

Most of the data is available at https://www.who.int/teams/social-determinants-of-health/safety-and-mobility/global-report-on-drowning-prevention (and more specifically in the WHO Drowning Prevention Data app); other information might be shared upon request to corresponding author.

## References

[1] World Health Organization. Global status report on drowning prevention 2024. Geneva, World Health Organization.: 2024. Licence: CC BY-NC-SA 3.0 IGO. Available from: https://iris.who.int/bitstream/handle/10665/379812/9789240103962-eng.pdf?sequence=1 (accessed on March 16, 2025).

[2] Pan American Health Organization. Leading causes of death and disease burden in the Americas: Noncommunicable diseases and external causes. Washington, D.C.: PAHO. 2024. Available from: 10.37774/9789275128626

[3] United Nations. Global Drowning Prevention [resolution 45/273]. 75th United Nations General Assembly; 29 April 2021. New York: United Nations. 2021. Available at: https://documents.un.org/doc/undoc/gen/n21/106/27/pdf/n2110627.pdf (accessed on March 16, 2025).

[4] World Health Organization. Accelerating action on global drowning prevention [resolution 76/18]. 76th World Health Assembly; 30 May 2023. Available at: https://apps.who.int/gb/ebwha/pdf_files/WHA76/A76_R18-en.pdf (accessed on March 16, 2025).

[5] World Health Organization. Global Report on Drowning. Geneva: World Health Organization 2014. Available from: https://iris.who.int/bitstream/handle/10665/143893/9789241564786_eng.pdf?sequence=1 (accessed on March 16, 2025).

[6] World Health Organization. Preventing drowning: an implementation guide. Geneva: World Health Organization 2017. Available from: https://iris.who.int/bitstream/handle/10665/255196/9789241511933-eng.pdf?sequence=1 (accessed on March 16, 2025).

[7] World Health Organization. Preventing drowning, practical guidance. Geneva: World Health Organization 2022. Available from: https://iris.who.int/bitstream/handle/10665/352699/9789240046726-eng.pdf?sequence=1 (accessed on March 16, 2025).

[8] Queiroga AC, Pérez-Núñez R, the first WHO Global Status Report on Drowning Prevention. Lancet Reg Health Am. Drowning prevention in the Americas: current and future opportunities arising from. 2023; 26:100585.10.1016/j.lana.2023.100585. eCollection 2023 Oct. Available from: https://www.thelancet.com/action/showPdf?pii=S2667-193X%2823%2900159-X (accessed on March 16, 2025). 10.1016/j.lana.2023.100585 PMC10493580 37701458

[9] The Lancet. Anyone can drown. No one should. Lancet Global Health. 2024. Available from: https://www.thelancet.com/action/showPdf?pii=S2468-2667%2824%2900305-0 (accessed on March 16, 2025).

[10] Pérez-Núñez R, Vera-López JD. Las asfixias accidentales En méxico: Un problema de Salud pública oculto. Gac Sanit. 2020;34(6):572–81. 10.1016/j.gaceta.2019.05.003. 31300326 10.1016/j.gaceta.2019.05.003

[11] Vera-López JD, Hidalgo-Solorzano E, Perez-Núñez R. Riesgos de accidentes En El hogar: factores asociados y Su efecto sobre La ocurrencia de accidentes En grupos vulnerables. Salud Publica Mex. 2022;64(2):196–208. 10.21149/12971. 35438926 10.21149/12971

[12] Báez-Báez GL, Orozco-Valerio M, Méndez-Magaña AC, Dávalos-Guzmán J, Celis A. Drowning risk factors into cistern for children 1 to 4 years old. Rev Invest Clin. 2014;66(1):24–30. 24762724

[13] Celis A. Home drowning among preschool age Mexican children. Inj Prev. 1997;3(4):252–6. 10.1136/ip.3.4.252. 9493619 10.1136/ip.3.4.252 PMC1067849

[14] World Health Organization. Hidden depths: the global investment case for drowning prevention. Geneva: World Health Organization. 2023. Licence: CC BY-NC-SA 3.0 IGO. https://iris.who.int/bitstream/handle/10665/371701/9789240077720-eng.pdf?sequence=1 (accessed on March 16, 2025).

[15] Ashraf L, Zia N, Vincenten J, Mackay JM, Agrawal P, Green A, et al. Effectiveness of interventions to prevent drowning among children under age 20 years: a global scoping review. Front Public Health. 2024;12:1467478. 10.3389/fpubh.2024.1467478. 39811781 10.3389/fpubh.2024.1467478 PMC11729736

